# The impact of acute changes of inflammation on appetite and food intake among older hospitalised patients

**DOI:** 10.1017/S0007114520002160

**Published:** 2020-11-28

**Authors:** Maryam Pourhassan, Lars Sieske, Gregor Janssen, Nina Babel, Timm Henning Westhoff, Rainer Wirth

**Affiliations:** 1 Department of Geriatric Medicine, Marien Hospital Herne, Ruhr-Universität, 44625 Bochum, Germany; 2 Medical Department I, General Internal Medicine, Marien Hospital Herne, Ruhr-Universität, 44625 Bochum, Germany

**Keywords:** Appetite, Cachexia, Food intake, Inflammation, Malnutrition, Older subjects

## Abstract

The present study aimed to investigate the effect of acute changes in serum C-reactive protein (CRP) on appetite and food intake among older hospitalised patients. A total of 200 patients (age range 65–94 years, 62·5 % women) participated in this prospective longitudinal observational study. Risk of malnutrition was measured according to the Mini Nutritional Assessment Short Form. The Simplified Nutritional Appetite Questionnaire (SNAQ) and Edmonton Symptom Assessment System (ESAS) were used to evaluate patients’ appetite at the time of hospital admission (baseline) and after 7 d (follow-up). Food intake was measured according to the plate diagram and serum CRP was analysed at baseline and follow-up. At baseline, 30·5 % of the patients had moderate to severe inflammation, 31·0 % were malnourished and 48·0 % had food intake <75 % of the meals offered. Also, 32·5 and 23·5 % reported poor and very poor appetite or severe loss of appetite according to the SNAQ and ESAS, respectively. Of the patients, 40 % displayed a pronounced reduction in median CRP levels by −1·2 mg/dl and 19 % demonstrated an increase in median CRP levels by +1·2 mg/dl. Appetite significantly improved (*P* = 0·006) in patients with a decrease in CRP level and deteriorated in those with an increase in CRP level (*P* = 0·032). Changes in CRP levels did not show any significant impact on food intake. In a regression analysis, changes of inflammation were the major independent predictor for changes of patients’ appetite. We conclude that inflammation has a significant impact on appetite and should therefore be considered in the diagnosis and treatment of malnutrition.

Older individuals frequently experience negative health-related outcomes, such as decline in physical and cognitive functioning, psychological disorders and acute and chronic diseases^(1)^. Some of these factors may cause metabolic changes, suppress appetite and impair food consumption leading to the development of malnutrition^(2,3)^. Malnutrition is associated with frailty, diminished quality of life, longer hospital stays, morbidity and mortality^(4)^. Although, the prevalence of malnutrition is as high as 50·0 % in older hospitalised patients^(5,6)^, it remains frequently unrecognised^(7)^ and the pathophysiological role and relevance of potential causes are still unclear^(8,9)^.

Numerous factors have been identified as potential causes of malnutrition^(9,10)^, with inflammation suspected to be one of the major factors. Indeed, clinical evidence demonstrated that malnutrition and inflammation are interrelated which led to the term malnutrition–inflammation–cachexia syndrome^(11–13)^. During acute illness with inflammation, energy requirements increase which may *per se* result in disease-related malnutrition.

However, inflammatory response may also reduce appetite and alter feeding behaviour^(14)^. Thus, the relationship between inflammation, appetite and food intake is not well studied. Association between decreased appetite or food intake and elevated levels of inflammation, as marked by increased C-reactive protein (CRP), have been previously reported in dialysis^(15–17)^, cancer^(18)^ and geriatric patients^(19,20)^. Findings of a study in 240 intensive care unit (ICU) survivors with median age of 62 years (interquartile range (IQR) 52–70 years) demonstrated a significant association of CRP concentration and appetite at 1 week after ICU discharge but not at 3 months after ICU discharge^(21)^. Another study among 445 older patients (age range 70–81 years) indicated that patients with acute-phase response as measured by CRP > 1 mg/dl had lower energy intake in hospital, whereas nutritional status improved in those without acute-phase response^(3)^.

However, much of the evidence regarding the associations of inflammation with appetite and food intake were constricted to cross-sectional studies. Although longitudinal studies cannot prove causality, the association of concomitant changes of inflammation and appetite may substantiate the probable causal relationship. To the best of our knowledge, there is no data available examining the role of inflammation on appetite and food in a prospective longitudinal study in older hospitalised patients. Therefore, the present study aimed to investigate the effect of acute changes in serum CRP, as an inflammatory marker, on appetite and food intake among older hospitalised patients.

## Subjects and methods

This prospective longitudinal observational study was undertaken between September 2017 and November 2018 at a geriatric acute care unit, at the university hospital, Marien Hospital Herne in Germany. The study participants comprise 200 consecutive older hospitalised individuals with mean age 81·4 (sd 6·6) years. Inclusion criteria were age ≥65 years, a probable hospital stay of at least 7 d, ability to understand and cooperate and written informed consent. Participants with suspected or diagnosed dysphagia, paralysis, severe cognitive impairment (Montreal Cognitive Assessment (MoCA) <10) and artificial nutrition, that is, tube feeding and parenteral nutrition were excluded from the study. Body weight, nutritional status and geriatric assessment were conducted at the first days after hospital admission. Measurements of food intake, appetite and inflammation were made at time of the hospital admission or the following day (baseline) and after 7 d (follow-up). The present study was conducted according to the guidelines laid down in the Declaration of Helsinki, and all procedures involving human patients were approved by the ethical committee of Ruhr-University Bochum (no. 16-5956, approved on 4 April 2017). Written informed consent was obtained from all patients.

### Geriatric assessment

Risk of malnutrition was measured according to the Mini Nutritional Assessment Short Form^(22)^ and participants were stratified as normal nutritional status (12–14 points), at risk of malnutrition (8–11 points) or malnourished (0–7 points). Frailty was diagnosed based on the FRAIL scale^(23)^ with score 0 being not frail, 1–2 pre-frail and 3–5 frail. The Barthel index (BI) was used to assess self-caring activities^(24)^. The points’ range of the German version of the BI is 0–100 points, with 100 points indicating independence in all activities of daily living. The MoCA^(25)^ was used to evaluate cognitive function with a total score of 30, whereas a score of 26 and higher considered normal. Depressive symptoms was diagnosed using the Depression in Old Age Scale^(26)^ with scores 0–2 as having no depression, 3 suspected depression and 4–10 probable depression. The risk of sarcopenia was determined based on the SARC-F questionnaire^(27)^ with a total score of 10, and subjects with score ≥4 were defined as having probable sarcopenia. The Charlson comorbidity index (CCI)^(28)^ was used to determine medical co-morbidities.

### Assessment of appetite and food intake

#### Appetite

The Simplified Nutritional Appetite Questionnaire (SNAQ)^(29)^ and Edmonton Symptom Assessment System (ESAS)^(30)^ were used to evaluate patients’ appetite at baseline and follow-up. The SNAQ comprises four domains, including appetite, feeling of fullness, taste of food and meal frequency, scaling from 1 (worst possible) to 5. Based on the respective SNAQ-appetite question, patients were grouped into three categories as having very poor and poor (score 1–2), average (score 3) and good and very good appetite (score 4–5). The ESAS is composed of nine pre-defined symptom domains, including pain, tiredness, nausea, depression, anxiety, drowsiness, appetite, well-being, shortness of breath and an optional tenth symptom, which can be defined by the examiner. Each item yields scores ranges from 0 (no symptom) to 10 (worst possible severity). Using the respective ESAS-appetite item, patients with scores 0–3 and scores 4–6 were considered as having no and moderate loss of appetite, respectively, whereas subjects with score ≥7 were defined as severe loss of appetite.

#### Food intake

The semi-quantitative plate diagram method^(31)^ was used to determine food intake at baseline and follow-up. After each main meal, a senior nurse estimated and recorded the proportion of patients’ food intake of the meal served for 1 d (0, 25, 50, 75 or 100 %). Snacks between main meals and oral nutritional supplements were not considered. The recorded percentage of all three main meals were summed and divided by three to derive estimation of average food intake over 1 d. Thereafter, patients were grouped into two categories as food intake <75 and ≥75 % of meals served.

### Measurement of C-reactive protein

Blood samples for analysing serum CRP were obtained at two time points corresponding to the first appetite assessment and food intake and after 7 d. According to our laboratory, CRP levels <0·5 mg/dl are considered as normal. However, patients were further categorised according to their CRP levels to differentiate mild inflammation from severe inflammation. A CRP level greater than 3·0 mg/dl is considered as moderate to severe inflammation^(32)^, while CRP levels between 0·5 and 3·0 mg/dl are defined as mild inflammation. In addition, CRP levels changes within a 40·0 % range were considered as no change, whereas a decrease or increase >40·0 % of initial CRP levels were considered as a significant change in CRP^(33)^.

### Statistical analysis

All statistical analyses were performed using SPSS statistical software (SPSS Statistics for Windows, IBM Corp., version 24.0). With the expected mean values of eleven-point ESAS-appetite scale (0–10) in group of patients with inflammation (mean value 4) and without inflammation (mean value 6), a case number of 200 in 1:1 design with a power of 0·8 and a type I error of 0·05 is calculated (www.clinical-trials.de).

Means and standard deviations were used for continuous data with normal distribution, whereas median values are expressed with IQR for non-normally distributed data. Categorical variables are shown as absolute numbers and percentages (*n*, %). To explore the association of appetite and food intake with inflammation at the time of admission and after 7 d, we performed the Kruskal–Wallis test followed by pairwise comparison or *χ* ^2^ test as appropriate. In addition, differences in appetite and food intake from baseline to follow-up across categories of CRP levels were tested using the Wilcoxon signed rank test. Differences in mean CRP over time were analysed using the paired samples *t* test. Moreover, the Wilcoxon–Mann–Whitney test was performed to examine the association of patients’ appetite and food intake. Multiple regression analysis was used to determine the impact of risk factors (i.e. changes in CRP levels, infection, chronic inflammatory diseases, sex and age as independent variables) on changes in patients’ appetite (as dependent variable). Statistical significance was set at *P* < 0·05.

## Results

### Characterisation of study population

Baseline characteristics of study participants are described in Table 1, and a detailed description of the baseline data has been reported elsewhere^(20)^. Briefly, 62·5 % of subjects were women and 37·5 % of subjects were men. The age range was between 65 and 98 years. Major diagnoses defined as reason for hospital admission were falls and fractures, pneumonia, osteoarthritis, post-stroke care and urinary tract infection. On admission, 15·5 % of the patients had a history of acute infections such as pneumonia and urinary tract infection, whereas 11·0 % had a chronic inflammatory disease such as rheumatoid arthritis, chronic hepatitis or chronic obstructive pulmonary disease. In the total population, 31·0 % of the patients were malnourished and 42·0 % displayed severe depressive symptoms. In addition, 81·0 % of the participants were frail and nearly the same proportion had probable sarcopenia (82·0 %) according to SARC-F score. The majority of the subjects were mildly or moderately cognitively impaired (93·0 %).

**Table 1.** Characteristics of the study population at baseline (Numbers and percentages; mean values and standard deviations; medians and interquartile ranges (IQR))

	Total population (*n* 200)		
	*n*		%
Sex			
Female	125		62·5
Male	75		37·5
Age (years)			
Mean		81·4	
sd		6·6	
Height (m)			
Mean		1·66	
sd		0·08	
Actual body weight (kg)			
Mean		73·2	
sd		17·9	
BMI (kg/m^2^)			
Mean		26·5	
sd		6·5	
MNA-SF			
Median		9	
IQR		7–10	
Normal nutritional status	17		9·0
At risk of malnutrition	119		60·0
Malnourished	61		31·0
Barthel index on admission			
Median		45	
IQR		35–55	
FRAIL simple scale score			
Median		4	
IQR		3–4	
SARC-F scores			
Median		6	
IQR		4–8	
Cognitive function (MoCA)			
Median		20	
IQR		16–23	
Depression score (DIA-S)			
Median		3	
IQR		1–5	
Charlson comorbidity index			
Median		3	
IQR		2–4	
Infection on admission			
Yes	31		15·5
No	169		84·5
Chronic inflammatory diseases			
Yes	21		11·0
No	166		89·0

MNA-SF, Mini Nutritional Assessment Short Form (normal nutritional status 12–14 points, at risk of malnutrition 8–11 points and malnourished 0–7 points); FRAIL simple scale (not frail with score 0, pre-frail with scores of 1–2 and frail with scores of 3–5); SARC-F scores (high risk of sarcopenia with score ≥4); MoCA, Montreal Cognitive Assessment (scores <26 considered as cognitively impaired); DIA-S scores, Depression in Old Age Scale (no depressive symptom with 0–2 points, suspected depression 3 point and probable depression 4–10 points).

### Changes in inflammation, appetite and food intake

Results of changes in CRP levels, appetite and food intake from baseline to follow-up are shown in Table 2. At baseline, 30·5 % of the patients had moderate to severe inflammation and 48·0 % had food intake <75 % of the meals offered. In addition, 32·5 and 23·5 % reported poor and very poor SNAQ-appetite or severe loss of ESAS-appetite, respectively.

**Table 2.** Changes in inflammation, appetite and food intake from baseline to follow-up in total population (*n* 200) (Numbers and percentages; mean values and standard deviations; medians and interquartile ranges (IQR))

	Baseline			Follow-up			*P*
	*n*		%	*n*		%	
CRP (mg/dl)							
Mean		3·5			2·7		
sd		5·7			4·2		
No inflammation	25·5		51	32·5		65	
Mild inflammation	44·0		88	41·5		83	0·012
Moderate to severe inflammation	30·5		61	26·5		52	
SNAQ score							
Median		14			14		
IQR		12–15			12–16		
<14	43·5		87	40·5		81	0·363
≥14	56·5		113	59·5		119	
SNAQ-appetite							
Median		3			3		
IQR		2–4			3–4		
Very poor and poor	32·5		65	23·5		47	
Average	36·0		72	42·0		84	0·043
Very good and good	31·5		63	34·5		69	
ESAS-appetite							
Median		2			3		
IQR		0–6			0–5		
No loss of appetite	56·0		112	55·0		110	
Average loss of appetite	20·5		41	25·5		51	0·692
Severe loss of appetite	23·5		47	19·5		39	
Food intake*							
Median		75			67		
IQR		50–83			50–92		
<75 % of intake	48·0		95	52·0		103	0·219
≥75 % of intake	52·0		102	48·0		94	

CRP, C-reactive protein (no inflammation 0–0·499 mg/dl, mild inflammation 0·5–3·0 mg/dl and moderate to severe inflammation >3·0 mg/dl); SNAQ score, Simplified Nutritional Appetite Questionnaire (maximum score 20, score <14 indicates risk of at least 5 % weight loss within 6 months); SNAQ-appetite rated from very poor and poor (1 and 2 points), average (3 points) and good and very good (4 and 5 points); IESAS, Edmonton Symptom Assessment System; ESAS-appetite categories as no (0–3 points), average (4–6 points) and severe (7–10 points) loss of appetite.

* Food intake was measured according to the plate diagram.

In total population, mean CRP significantly decreased by −0·8 (sd 4·6) mg/dl (*P* = 0·012) and appetite significantly improved (*P* = 0·043) from baseline to follow-up, whereas total SNAQ score and food intake remained unchanged over time (Table 2).

Of the study population, 40 % (*n* 80) displayed a pronounced reduction in median CRP levels (>40 % of baseline value) by −1·2 (IQR –3·7 to –0·40) mg/dl and nearly the same proportion (*n* 82, 41·0 %) kept their CRP levels of baseline values (median change in CRP –0·10 (IQR –0·50 to +0·10) mg/dl). Of the patients, 19 % (*n* 38) demonstrated an increase in CRP levels (median change in CRP +1·2 (IQR +0·57 to +3·5) mg/dl).

At baseline, there were no statistically significant differences across CRP levels with regard to demographic characteristics, nutritional status and geriatric assessment except for mean CRP (*P* < 0·001) and infection (*P* < 0·001). There were significant negative associations between CRP levels and SNAQ-appetite and food intake at baseline (*P* = 0·003 and *P* < 0·001, respectively) and at follow-up (*P* = 0·050 and *P* = 0·004, respectively). Similar associations at both times were observed after excluding patients with acute infection. In addition, significant negative associations between ESAS-appetite and nutritional status (*P* = 0·026) were found, whereas malnourished older patients demonstrated a severe loss of appetite compared with those with normal nutritional status (*P* = 0·011). Moreover, mean CRP was almost two times higher in patients with food intake <75 % of meal compared with those with food intake ≥75 % at baseline (4·9 (sd 7·3) *v*. 2·1 (sd 3·1) mg/dl, respectively; *P* < 0·001) and follow-up (3·4 (sd 5·1) *v*. 1·8 (sd 2·7) mg/dl, respectively; *P* = 0·01). Further, significant positive associations between food intake and SNAQ-appetite at baseline (*P* < 0·001) and follow-up (*P* < 0·001) were observed.

### Impact of inflammation changes on appetite and food intake

Table 3 shows the association between changes in CRP levels and changes in appetite and food intake overtime. Appetite significantly improved (*P* = 0·006) in the group of patients with a pronounced decrease in CRP level and remained unchanged over time in those with no change in CRP levels. In contrast, increase in CRP levels from baseline to follow-up was associated with deterioration in appetite (*P* = 0·032). In a regression analysis, changes of inflammation (CRP) were the major independent predictor for changes of patients’ appetite (*P* = 0·047), whereas other variables such as infection (*P* = 0·081), chronic inflammatory diseases (*P* = 0·247), age (*P* = 0·226) and sex (*P* = 0·592) did not show any impact on appetite changes. In addition, changes in CRP levels did not demonstrate any significant effect on food intake. When compared with patients with appetite improvement, patients with deterioration in appetite had a significantly lower food intake than before (*P* = 0·006).

**Table 3.** Association between changes in CRP levels and changes in appetite scores and food intake from baseline to follow-up in total population (*n* 200)

	*P*		
	Change in SNAQ-appetite score	Change in ESAS-appetite score	Change in food intake*
Decreased in CRP levels (*n* 80)	0·006	0·662	0·726
No change in CRP levels (*n* 82)	0·421	0·092	0·354
Increase in CRP levels (*n* 38)	0·507	0·032	0·116

CRP, C-reactive protein (no inflammation 0–0·5 mg/dl, mild inflammation 0·5–3·0 mg/dl and moderate to severe inflammation >3·0 mg/dl); SNAQ, Simplified Nutritional Appetite Questionnaire; ESAS, Edmonton Symptom Assessment System.

* Food intake was measured according to the plate diagram.

## Discussion

The present study has shown that appetite and food intake of older hospitalised patients are associated with inflammation, as measured by CRP, at the time of hospital admission and after 7 d. A relationship between inflammation and appetite and food intake has been previously shown in haemodialysis^(15,34)^ and advanced cancer patients^(18)^. However, few published studies have investigated this association among an older population^(3,19)^ and causality remains unclear.

Findings of a cross-sectional pilot study among older hospitalised patients (mean age 83·1 (sd 6·5) years) indicated the significant impact of higher CRP levels on low food intake^(19)^. In addition, another study among older hospitalised patients, Gariballa *et al*.^(3)^ reported that higher levels of CRP were associated with low food intake during hospital, but in both studies appetite was not measured. Despite growing interest in understanding the role of inflammation in development of malnutrition, the knowledge regarding this issue is scarce and the evidence is mainly limited to cross-sectional studies and food intake, not appetite. To our knowledge, this is the first study investigating the impact of inflammation changes on appetite and food intake in older hospitalised patients.

A major finding of the present study is that significant changes in inflammation from baseline to follow-up had a potential impact on appetite. Namely, appetite significantly improved in the group of patients with reduction in CRP levels and significantly deteriorated over time in those with an increase in CRP level. In accordance with our study, Merriweather *et al*.^(21)^ found a significant improvement in median appetite score during the first 3 months after ICU discharge among ICU survivors (median age of 62 years), whereas CRP was significantly correlated with appetite at baseline but not at 3 months follow-up. However, the authors did not examine the effect of inflammation changes on appetite.

Older people are at increased risk of malnutrition due to functional, physiological and psychological issues coupled with concurrent medical problems^(2)^. Malnutrition and inflammation commonly coexist in older patients due to inflammaging^(35)^ and chronic and acute illnesses. Severe disease, which is associated with tissue inflammation, leads to increased levels of pro-inflammatory cytokines, such as IL1, IL6 and CRP that are thought to contribute to appetite deterioration^(36)^. Indeed, pro-inflammatory cytokines may be involved in negative energy balance, increased muscle breakdown and development of malnutrition^(3,15,16,37)^, but their role in suppressing appetite and food intake remains unknown. As a result, a vicious cycle of acute disease, inflammation and malnutrition may negatively affect overall health outcomes of older adults.

Normal nutrition and appetite are significant determinants of health in older individuals. In the present study, significant associations between appetite and food intake were observed. Namely, patients with poor and very poor appetite revealed a lower food intake at baseline and follow-up. More importantly, deterioration in appetite overtime was accompanied with lower food intake. In line with our findings, in a cross-sectional study of 2597 community-dwelling older individuals (mean age 74·5 (sd 2·8) years), van der Meij *et al*.^(38)^ reported that older adults with a poor appetite have a lower energy intake. The regulative role of inflammation and its effect on the central control of food intake and appetite are complex and not fully understood. However, the hypothalamus is considered being responsible for nutrient sensing, appetite behaviour and energy metabolism^(39)^. Indeed, the hypothalamus receives signals about the nutritional state via afferent signals from enteric nervous systems and several hormones. Recent studies demonstrate that inflammation, occurring either within enteric and brain tissue or systemically, plays an essential role in hypothalamic adaptation^(40,41)^.

It is worth mentioning that in the present study, mean CRP was almost two times higher in patients with food intake <75 % of the meals offered compared with those with food intake ≥75 % at baseline and follow-up. Nevertheless, despite changes in appetite subsequent to inflammation changes, inflammation alteration does not seem to have a similar impact on food intake in the present study. However, there were significant associations between inflammation and food intake at the time of admission and after 7 d. Our data suggested that the effect of inflammation changes on food intake is less prominent compared with appetite, indicating that alteration in food intake may need more time to be restored after decrease in inflammation. In addition, it can be hypothesised that improvement in food intake in older patients with systematic inflammation may be more efficacious after complete subsidence of inflammation. In our study, even in the group of patients with a reduction in CRP level, 37·0 and 13·0 % still have mild and moderate–severe inflammation, respectively. Further, the accurate collection of food consumption is a difficult task and some of the methods are expensive, time-consuming and are not easy to use^(31)^. However, the plate diagram is a validated tool for estimation of energetic intake but it can only roughly measure food intake. In a study among hospitalisd patients aged 19–94 years, Bjornsdottir *et al*.^(31)^ indicated that using a plate diagram, energy intake could be estimated with fair accuracy.

Several limitations to the present study bear mention. We used a heterogeneous group of geriatric hospitalised population. There is no assessment tool for evaluating appetite; however, we used a single question of the SNAQ and one scale of the ESAS, which is originally use for the assessment of palliative care patients. In addition, inflammatory markers, such as IL1, IL6 and TNF-*α* which are known to induce anorexia or may have stronger impact on appetite and food intake compared with CRP, were not measured in the present study. Aside from inflammation, loss of appetite and low food intake may be associated with other clinical risk factors rather than inflammation alone. Therefore, residual uncontrolled confounding cannot be excluded. Nevertheless, the association of concomitant changes of inflammation and appetite cannot proof but may substantiate causality. Further intervention studies focusing on inflammation should also take into account to what extent modification or subsidence of inflammation can improve appetite and nutritional intake in older adults.

### Conclusion

We conclude that inflammation has a significant impact on appetite and should be therefore considered in diagnosis and treatment of malnutrition.
